# Precision Rehabilitation After Youth Anterior Cruciate Ligament Reconstruction: Individualized Reinjury Risk Stratification and Modifiable Risk Factor Identification to Guide Late-Phase Rehabilitation

**DOI:** 10.1177/23259671251329355

**Published:** 2025-04-11

**Authors:** Elliot M. Greenberg, Amanda Watson, Kimberly Helm, Kevin Landrum, J. Todd R. Lawrence, Theodore J. Ganley

**Affiliations:** † Division of Orthopaedics, Children’s Hospital of Philadelphia, Philadelphia, Pennsylvania, USA; ‡ Perelman School of Medicine, University of Pennsylvania, Philadelphia, Pennsylvania, USA; § School of Engineering and Applied Science, University of Virginia, Charlottesville, Virginia, USA; ‖ School of Engineering and Applied Science, University of Pennsylvania, Philadelphia, Pennsylvania, USA; Investigation performed at the Children’s Hospital of Philadelphia, Philadelphia, Pennsylvania, USA

**Keywords:** anterior cruciate ligament, reinjury, pediatric, adolescent, rehabilitation, machine learning

## Abstract

**Background:**

After anterior cruciate ligament (ACL) reconstruction, adolescent athletes have a high risk of second ACL injuries, and revision ACL reconstruction is associated with increased medical costs, reduced activity levels, chronic knee pain, and higher rates of knee osteoarthritis, making the prevention of a reinjury a priority. While athlete clearance protocols and algorithms exist, the current methods of identifying the reinjury risk have limited predictive accuracy and are largely based on nonmodifiable risk factors, which limit their clinical application.

**Purpose:**

The goal of this study was to develop an ACL reinjury risk prediction (ACL-RRP) model capable of accurately classifying an individual patient’s risk, identifying modifiable risk factors, and ranking these factors in the order of importance and ability to be modified.

**Study Design:**

Cohort study (Diagnosis); Level of evidence, 2.

**Methods:**

A clinician-informed approach was utilized to develop the prediction model and an interpretable output system. The primary outcome variable was the likelihood of sustaining a repeat ACL injury. The data were split into training (80% [n = 628]) and holdout (20% [n = 158]) datasets to train and subsequently validate the model. The accuracy of classification was identified by the sensitivity, specificity, positive/negative predictive values, and odds ratio.

**Results:**

The final model included 33 predictor variables, 23 of which are modifiable. The model adjusted the weight of the risk classification and risk factors (predictor variables) on a case-by-case basis. The model demonstrated a sensitivity of 94% and a specificity of 76%. Patients classified as being high risk had 4.5 times the risk of repeat ACL injuries compared with those classified as being low risk.

**Conclusion:**

This clinician-informed ACL-RRP model demonstrated a high degree of accuracy when classifying patients as having a high or low risk of repeat ACL injuries and generated patient-specific modifiable risk factors to guide ongoing rehabilitation or patient education to achieve the goals of reducing the ACL reinjury risk.

Anterior cruciate ligament (ACL) injuries occur at a high rate within youth athletes, and data indicate that this incidence is increasing.^1,2,9,25,26^ Adolescent athletes have a high rate of reinjuries, with some studies indicating rates as high as 30%.^14,17,29,32^ Revision ACL reconstruction is associated with reduced activity levels, higher rates of chondral or meniscal damage, chronic knee pain, and higher rates of knee osteoarthritis, making the prevention of reinjuries very important.^11,23,34^ The high reinjury rate in younger patients raises significant concerns for the long-term health of these patients and has prompted further exploration to identify risk factors for a reinjury and design mitigation strategies.^32^

Currently, clinicians employ a combination of objective clinical, physical performance, and time-based criteria to determine an athlete’s readiness to return to sports. While specific risk factors for a reinjury and predictive models exist,^6,10,13-15,18,27,30^ these current methods of identifying an athlete’s risk demonstrate limited predictive accuracy.^28,33^ Although the problem of reinjuries is multifactorial, the lack of accuracy may be grounded in underlying weaknesses within the modeling and development strategies utilized. Current models tend to be limited in scope and fail to consider a significant number of variables that have been shown to relate to an ACL reinjury. Previous models have also utilized traditional statistical modeling, which is not capable of analyzing the complex mechanisms of the cause of sports injuries.^16^ In addition, current models are not individualized and tend to stratify the risk based on the ability to satisfy recommended discharge criteria as a group (ie, those able to meet all return-to-sports strength or functional performance criteria).^14,18^ While this information is useful and provides valuable insight into the reinjury risk, it is not always helpful because it is not specific to each individual patient and does not sufficiently assist the clinician to identify the reinjury risk for those with mixed data (ie, those who can pass some but not all strength or functional performance criteria). Moreover, the majority of existing predictive modeling analyses focus primarily on patient and surgical variables that cannot be altered during the postoperative course, limiting the clinical utility and impact of their application.

Fostered by an era of improved data availability and enhanced machine learning capabilities, sports medicine researchers have been advocating for the adoption of artificial intelligence (AI) within sports injury research.^4^ Machine learning is a branch of AI that utilizes historical data to predict future clinical outcomes through computational algorithms capable of combining related factors and eliminating potential confounding factors to improve the accuracy of prediction.^31^ Compared with conventional statistical methods, such as regression analyses, machine learning is superior in simultaneously analyzing multiple predictive variables with their combinations and interactions rather than validating the a priori–assumed relationships between variables.^20,31^ Thus, machine learning provides opportunities to overcome the shortcomings of traditional statistical modeling in sports medicine by analyzing the complex interactions that occur between variables and allowing for the use of larger, more robust datasets.^4,16,22^

While promising, 2 frequently cited obstacles to instituting the widespread use of machine learning in medicine are a lack of available data to train these models and a lack of clarity or understanding about how machine learning models arrive at their decisions.^5^ To combat these issues, engineers have been advancing efforts on integrating machine learning with human knowledge and developing explainable AI methodologies. Integrating human knowledge into machine learning offers several advantages over traditional methods, including reduced data requirements and the ability to add clarity to the process, making machine learning decisions understandable to humans or, in our scenario, the sports medicine clinician.^7^ In addition, explainable AI techniques allow for the capacity to understand the model’s behavior (1) as a whole (eg, What is the most significant risk factor for ACL injuries within the entire cohort?) or (2) on an individual level (eg, Why is this particular patient at risk of an ACL injury?). Thus, the use of explainable AI within injury prediction models can not only offer clinicians the predicted output but also allow for the visualization of the effect that specific variables have on a patient’s risk classification.

The purpose of this study was to describe the development and accuracy of an ACL reinjury risk prediction (ACL-RRP) model using clinician-informed, explainable AI machine learning methods that is capable of distinguishing patients at a high risk of sustaining a subsequent ACL injury. Further, we aimed to demonstrate the use of a novel feedback application that identifies individualized modifiable risk factors that can be altered during rehabilitation to reduce the predicted risk.

## Methods

### Study Design

This was a retrospective case-control study approved by the institutional review board of the Children’s Hospital of Philadelphia.

#### Dataset

This dataset was derived from a single tertiary-care children’s hospital and represents a targeted subset extracted from a larger database of patients who underwent ACL reconstruction from 2009 to 2016. To qualify for inclusion in this study, participants had to have undergone isolated ACL reconstruction, with or without meniscus involvement. An ACL graft injury or contralateral ACL injury was determined by 1 of 2 methods: (1) an electronic medical record indicating that a clinical follow-up for a reinjury had occurred or (2) the patient was contacted by telephone or email and filled out a survey indicating a reinjury. Patient contact for the reinjury survey occurred at the beginning of 2017, resulting in a follow-up period that ranged from 1 to 7 years after surgery. Participants were excluded if they had undergone multiligament surgery, had chondral damage that required additional surgical interventions, or had undergone revision ACL reconstruction. Included participants were randomly divided into 2 distinct datasets: a training dataset (80%) used to develop the model and a holdout dataset (20%) that contained novel data not utilized in model development but later used for validation purposes. The 80/20 data allocation is standard practice in machine learning, creating a dataset that is robust enough to train the model and leaving adequate data to make reliable assessments of the model’s validity and generalizability. The data were randomly assigned into either training or validation using a custom algorithm designed to randomize allocation while keeping consistent the distribution of age, sex, and reinjury between groups. All variables within the dataset were considered for analysis and included information related to demographic characteristics, surgical techniques and details, familial history of ACL injuries, sports participation, and lower extremity strength and functional hop test results.

#### Missing Data and Imputation

There was a missing data rate of 12.1% (1177 missing data points of 9702 total). Multiple imputation methods including mean, integer, k-nearest neighbors, and decision tree were implemented and compared regarding the resulting effect on model performance while limiting the potential for overfitting of the model to the original dataset. The final list of predictor variables is provided in Table 1.

**Table 1:** Predictor Variables Included in Model Development^*a*^

Variable
Strength testing^*b*^
Involved limb hamstring normalized to body weight^*c*^
Involved limb quadriceps normalized to body weight^*c*^
Uninvolved limb hamstring normalized to body weight^*c*^
Uninvolved limb quadriceps normalized to body weight^*c*^
Peak torque quadriceps LSI^*c*^
Involved limb quadriceps total work^*c*^
Total work quadriceps LSI^*c*^
Total work hamstring LSI^*c*^
Ratio hamstring/quadriceps (involved)^*c*^
Ratio hamstring/quadriceps (uninvolved)^*c*^
Family history
First-degree relative with history of ACL injuries
Any relative with history of ACL injuries
Demographic characteristics
Sex
Age at time of surgery
Age at time of return to sports
Body mass index^*c*^
Functional hop testing
Single hop LSI^*c*^
Triple hop LSI^*c*^
Crossover triple hop LSI^*c*^
Timed hop LSI^*c*^
Vertical hop LSI^*c*^
Timed hop performance (involved limb)^*c*^
Triple hop normalized to height (involved limb)^*c*^
Vertical hop normalized to height (involved limb)^*c*^
Difference between triple and crossover hop (involved limb)^*c*^
Difference between triple and crossover hop (uninvolved limb)^*c*^
Surgical factors
Time from injury to surgery
Meniscal tear
Meniscal resection
Graft size
Patient-specific environmental factors
Time to release to full activity^*c*^
Sport played at time of initial ACL injury^*c*,*d*^
Time from surgery to repeat ACL injury

a Functional hop data were collected in the same time frame as strength data. ACL, anterior cruciate ligament; LSI, limb symmetry index.

b Strength was assessed using an isokinetic dynamometer at 180 deg/s at a mean of 299.1 ± 128.2 days after surgery.

c Modifiable risk factors.

d Sport played at time of initial injury was used as a proxy for sport played at time of repeat injury.

### Model Creation

#### Model Selection

The primary outcome was the likelihood of either an ACL graft injury or contralateral ACL injury. In total, 6 different machine learning approaches were applied and evaluated during model creation including (1) naïve Bayes, (2) k-nearest neighbors, (3) support vector machine, (4) decision tree, (5) random forest, and (6) extreme gradient boosting. A detailed review of each of these approaches is beyond the scope of the current article; however, each utilizes a different methodology to classify outcomes, and performance will vary based on the approach.^3^ Each technique was evaluated with regard to the sensitivity and specificity for predicting a repeat ACL injury.

#### Clinician-Informed Labeling Functions

**Figure 1.**
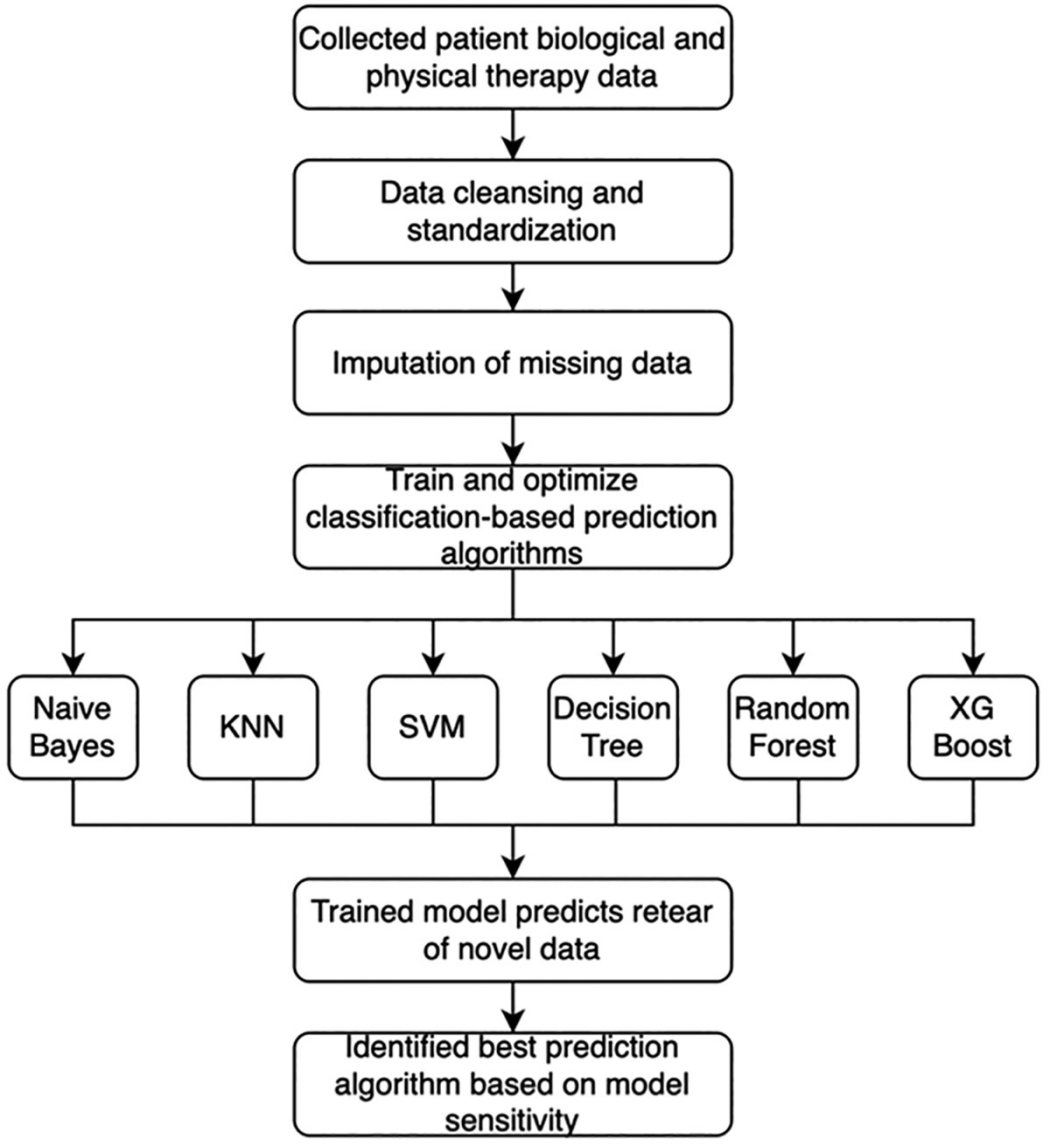
Model creation flow chart.

The creation of labeling functions is a process in which knowledge from clinicians is leveraged to enrich the capacity for prediction in machine learning programs. Simply stated, these labeling functions allow clinical insights to be used by the model to make better predictions. As such, each labeling function works with the machine learning algorithm and is evaluated for the ability to enhance the model’s predictive capability. Domain knowledge was obtained from 2 orthopaedic surgeons (J.T.L., T.G.) and 1 physical therapist (E.G.), who are all highly experienced in pediatric ACL reconstruction and rehabilitation. Each expert independently selected features or identified combinations of features that would be known as risk factors based on known literature and experience. These clinicians then produced a set of labeling functions for each risk factor, stratifying between high, low, and unknown risk (Appendix). These outputs were used by the model to predict a high risk or low risk based on these labeling functions after they had been applied to the data. The process for selecting the most successful model is shown in Figure 1.

#### Stratification of Modifiable Versus Nonmodifiable Risk Factors

To maximize the clinical utility of the ACL-RRP model, the outputs were differentiated between modifiable and nonmodifiable features or risk factors and ranked based on their ease of modification. The same clinicians (J.T.L., T.G., and E.G.) independently classified these risk factors as modifiable or nonmodifiable and then, for the risk factors deemed to be modifiable, ranked the “ease of modification” using a Likert scale, with 1 being easiest and 5 being most difficult to change.

#### Model Evaluation

Model accuracy was evaluated using 5-fold cross-validation and further validated utilizing the 20% holdout dataset. The diagnostic accuracy for a high risk of ACL reinjury was evaluated using the odds ratio, sensitivity, and specificity. In addition, the Youden index was calculated to reflect the diagnostic accuracy of the model for identifying those at risk of a repeat ACL injury while balancing for both the sensitivity and specificity.

## Results

The dataset consisted of 786 patients with a mean age of 15.1 ± 2.2 years and a female proportion of 47.8%. The demographic characteristics of the training and holdout datasets are outlined in Table 2. The dataset had an overall reinjury rate of 18.8%, with 86 ipsilateral and 62 contralateral ACL injuries, and the mean time to a reinjury was 704 days. The length of follow-up ranged from 1 to 7 years.

**Table 2:** Demographic Characteristics^*a*^

	Training Data (n = 628)	Holdout Data (n = 158)
Age, y	15.1 ± 2.2	15.1 ± 2.3
Female sex	305 (48.6)	71 (44.9)
Height, cm	166.5 ± 10.8	165.8 ± 11.9
Weight, kg	64.9 ± 16.9	64.8 ± 17.2
Time from injury to surgery, d	76.3 ± 98.5	76.9 ± 121.2
Time from surgery to return to activity, d	311.9 ± 92.9	320.9 ± 103.3
Time from surgery to reinjury, d	723.4 ± 503.1	684.6 ± 565.7
Type of graft		
Autograft	536 (85.4)	143 (90.5)
Allograft	25 (4.0)	4 (2.5)
Hybrid	7 (1.1)	3 (1.9)
Missing	60 (9.6)	8 (5.1)
Reinjury	120 (19.1)	28 (17.7)

a Data are shown as mean ± SD or n (%).

### Model Performance

#### Evaluation of Training Data

Model performance was evaluated using a combination of the various imputation methods and modeling algorithms (Table 3). Overall, naïve Bayes using mean as an imputation method was the highest performing model with a sensitivity of 94.0%, a specificity of 75.7%, and a Youden index of 0.697 (Table 3). The results from the cross-validation are shown in Table 4. Overall, the model remained stable across all folds of the cross-validation process, with the sensitivity ranging from 93.3% to 95.1%, the specificity ranging from 71.3% to 75.7%, and the Youden index ranging from 0.659 to 0.704.

**Table 3:** Results From Examined Machine Learning Models

	Imputation	Sensitivity	Specificity	Youden Index
Naïve Bayes	Mean	0.940	0.757	0.697
k-nearest neighbors	Integer	0.575	0.961	0.536
Decision tree	k-nearest neighbors	0.625	0.927	0.552
Extreme gradient boosting	k-nearest neighbors	0.417	0.990	0.407
Support vector machine	Decision tree	0.533	0.941	0.484
Random forest	Mean	0.416	0.990	0.406

**Table 4:** Cross-validation Results

Fold	Sensitivity	Specificity	Youden Index
1	0.936	0.757	0.693
2	0.933	0.751	0.684
3	0.946	0.713	0.659
4	0.951	0.753	0.704
5	0.936	0.742	0.678

#### Evaluation of Holdout Data

The evaluation of model accuracy within the holdout data demonstrated the prediction classification to be highly accurate and stable when analyzing novel data, indicating good generalizability. The ACL-RRP model showed a sensitivity of 94.3%, a specificity of 71.5%, and a Youden index of 0.657. Patients identified to be high risk within the holdout evaluation were 4.5 times (odds ratio, 4.5 [95% CI, 3.3-5.7]) more likely to sustain a repeat ACL injury than patients classified as low risk, with a sensitivity of 94.3% and a specificity of 71.5%.

#### Feedback System

**Figure 2.**
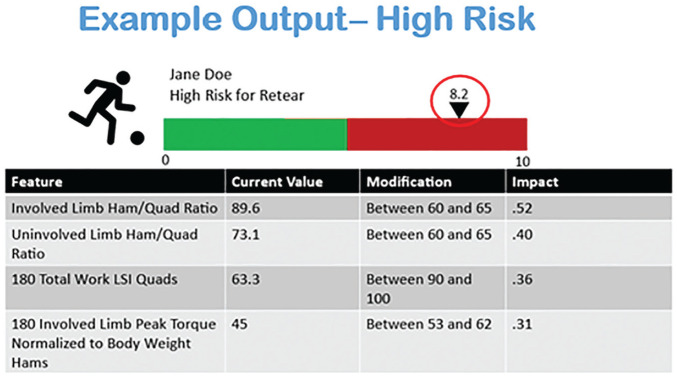
System output patient example. An example of a patient identified as being high risk from the holdout dataset and individual outputs from the anterior cruciate ligament reinjury risk prediction (ACL-RRP) model indicating the risk level (red circle) and modifiable risk factors ranked in order of importance. Providers would use these data to develop additional treatment plans to address the risk factors identified. As risk factors are addressed, the effect of each would be subtracted from the patient’s risk profile, and the ACL-RRP model would reanalyze that patient’s risk profile to determine the overall reduction in risk and if any new risk factors now contribute to the risk.

The system propagated an output illustrating the patient’s risk assessment on a graphical display and assigned an overall risk score from 0 (no risk) to 10 (highest risk) (Figure 2). The risk value attributed to nonmodifiable risk factors is displayed as well as the risk value associated with each of the modifiable risk factors deemed most responsible for elevating that patient’s risk. To assist the physician and the patient with decision making, risk factors are displayed and ranked based on their contribution to the reinjury risk and effect on risk reduction if they were modified.

## Discussion

The results of this study demonstrated successful achievement of developing an algorithm that can accurately distinguish between patients at a high risk and low risk of sustaining an ACL reinjury and offer specific individualized feedback to the clinician to guide ongoing clinical decision making. The ACL-RRP model incorporated and leveraged domain knowledge from clinicians and machine learning techniques to identify an individual patient’s risk for sustaining an ipsilateral graft rupture or contralateral ACL injury after ACL reconstruction. Once the risk level has been determined, the model utilizes the encoded knowledge from clinicians to stratify the risk factors into those that are modifiable and nonmodifiable while also taking account the ease of modification. Finally, the ACL-RRP model identifies the relative weight or importance of each modifiable factor that contributes to the patient’s overall reinjury risk (Figure 2). This information will enable clinicians to design and implement more personalized rehabilitation as well as educational or other risk-reducing interventions and to have more informed conversations regarding the reinjury risk with patients.

**Figure 3.**
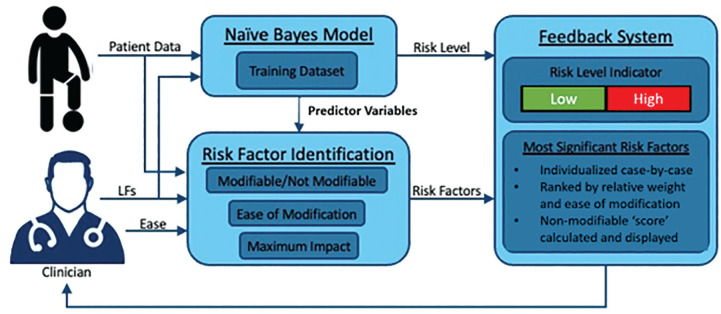
Schematic diagram of the anterior cruciate ligament reinjury risk prediction model showing the integrated process for model application, risk classification, and stratification of the feedback system, indicating the most significant patient-specific, modifiable risk factors ranked by impact on the risk and ease of modification. LF, labeling functions.

The entire system is composed of 3 components: the ACL-RRP model, the identification of risk factors, and the feedback system (Figure 3). The data-driven, personalized risk assessment carried out by the model allows clinicians to precisely tailor treatment recommendations and empower patients and families to make more informed treatment and activity choices after ACL reconstruction.

The unique development process of the ACL-RRP model offers several advantages over existing published machine learning applications evaluating the ACL reinjury risk. Martin et al^22^ analyzed the Norwegian National Knee Ligament Registry and utilized a Cox lasso model to develop an ACL revision risk calculator. While this national database provided many data points for machine learning development (24,935 patients), the dataset was imbalanced, with only 4.9% (n = 1219) of patients having a documented ACL reinjury. In addition, the model classified the majority of patients as being low risk. Similarly, Ye et al^35^ utilized an extreme gradient boosting model to predict ACL graft failure in a database of 432 patients. Within this dataset, only 3.7% (n = 16) of patients sustained an ACL graft injury. Thus, these datasets had a low prevalence of ACL reinjuries and were heavily biased toward patients who were either low risk or without a repeat ACL injury. This imbalance in outcomes data significantly limited the pool of available data for the development of an ACL reinjury algorithm, which may lead to inflated diagnostic accuracy and limit the generalizability of the results. In the development of the ACL-RRP model, the dataset contained a higher proportion of patients with a repeat ACL injury (18.8%), which ensured that there was an adequate number of reinjury data points available to develop stratification points between varying risk levels, improving the overall accuracy. These differences in data availability may be one reason that the ACL-RRP model has more stringent criteria or sensitivity in risk classification. Within the ACL-RRP model, patients deemed to be high risk were 45% more likely to sustain an ACL reinjury, while those classified as high risk in the study by Martin et al^22^ had a risk of only approximately 15% to 20%. In addition, the ACL-RRP model was developed exclusively within a dataset of pediatric and adolescent patients, which may allow a higher level of precision within this high-risk subset of the population. Given that the reinjury risk for adolescents can be as high as 30%,^32^ more stringent classification is necessary to ensure the clinical effect of these tools.

Numerous studies have identified a large number of risk factors for both primary and secondary ACL injuries, which include a variety of patient-specific (eg, demographic characteristics, skeletal anatomy), surgical (eg, graft size, fixation method), rehabilitation (eg, quadriceps strength, neuromuscular control), psychological (eg, fear of reinjuries, confidence), genetic, and environmental (eg, type of sports) features.^8,12,15,19,21,24,26^ While this diversity in potential risk factors makes an ACL injury an ideal environment to utilize machine learning applications, finding a data source rich enough in depth (number of participants) and breadth (number of predictor variables) is challenging and limits the clinical utility of the existing literature. Martin et al^22^ considered a total of 17 potential variables in their dataset, mostly constrained to demographic characteristics, preoperative patient-reported activity scale scores, and surgical/intraoperative techniques. The 5 predictor variables included within the final model were age, preoperative Knee injury and Osteoarthritis Outcome Score Quality of Life subscale score, graft choice, femoral fixation method, and time from injury to surgery. Similarly, Ye et al^35^ considered a total of 9 variables mostly within the same scope of those analyzed within Martin et al’s^22^ study, with additional factors related to intraoperative ratings of knee laxity and anatomy (ie, tibial inclination). The final variables included within Ye et al’s^35^ model were medial meniscal resection, participation in competitive sports, steep posterior tibial slope, and small graft diameter. In both cases, the predictor variables utilized in development were limited in scope, failing to consider several potential factors that may relate to the ACL reinjury risk and thus limiting the overall accuracy of the model. They also considered few, if any, modifiable risk factors that could potentially be addressed in the rehabilitation of these patients.

The breadth of variables available for consideration within the ACL-RRP model was a strength of this project. In total, 33 different variables representing demographic, surgical, environmental, strength, and functional performance factors were utilized in developing the ACL-RRP model. While these data are still limited in the overall scope of potential contributing factors, the strength of classification accuracy and the model for clinical application presented in this study show significant future promise as our work continues to grow. Our efforts on future model development focus on the expansion of predictor variables to include additional surgical, anatomic, psychological, neuromuscular control, quality of movement, and environmental factors. We hypothesize that the expansion of these predictor variables will further improve the model’s accuracy and clinical utility.

Being able to not only classify the ACL reinjury risk but also alter or affect one’s risk are critical steps for improving outcomes. To this point, developing a clinically relevant feedback system that would lead to actionable results was a priority in the formation of the ACL-RRP model. Figure 2 illustrates an example output for a patient included in model development. The model displays that specific patient’s overall risk on a scale from 0 to 10, with higher values indicating a higher risk of sustaining an ACL reinjury. The modifiable risk factors and their relative effect on the reinjury risk are displayed in a table format. This information offers the clinician clear talking points for patient education or family counseling regarding the reinjury risk while also providing specific insight into ongoing rehabilitation planning to reduce the risk. This individualized approach to risk determination and personalized modifiable risk factor identification is an essential step in moving toward an era of precision rehabilitation after ACL reconstruction.

Physical performance and postoperative rehabilitation data have been shown to have a significant effect on the ACL reinjury risk^14,18,27^; however, these data are limited within existing prospective registries and have not been widely included in machine learning approaches thus far. This fact indicates significant limitations within existing work, as performance data will likely significantly improve risk identification and represent clinical factors that would be most amenable to change during postoperative recovery. The inclusion of functional performance data in the ACL-RRP model may account for the higher level of risk stratification accuracy seen within our data compared with previously published reports that were not inclusive of these variables.^22,35^ This observation is further reinforced by the similarity of our results with previously published work demonstrating higher levels of risk assessment when rehabilitation variables are incorporated. Paterno et al^27^ performed a classification and regression tree analysis in a cohort of adolescent athletes who underwent ACL reconstruction, utilizing a similar set of surgical, demographic, and rehabilitation predictor variables. Their results identified 2 distinct profiles at a high risk of a repeat ACL injury in which data related to individual limb performance and limb symmetry on functional hop testing were common between both profiles. Differences in sample size, predictor variables, and machine learning approach may account for the higher sensitivity (94.0% vs 66.7%, respectively) seen within the current data compared with Paterno et al's^27^ study. Taken together, the high levels of risk identification seen within these studies and the high relative importance of hop performance within both studies demonstrate the value of including rehabilitation and physical performance data in ACL risk prediction.

### Limitations

This study is not without limitations. This was a retrospective study of young athletes who sustained a sports-related primary ACL injury, encapsulating a period from 2009 to 2016, and were from a single institution. While our dataset was large for most clinical research studies, it was still relatively small for machine learning applications. Participants within this study were willing to complete a prospective survey regarding their current reinjury or were identified as reinjured through a targeted extraction of patients who had a known ACL reinjury through presentation to our institution for reinjury treatment. While this targeted extraction allowed for a higher proportion of reinjured patients to balance our dataset and develop our model, this may bias our dataset because of the inability to capture all patients and outcomes. Similarly, while sports played at the time of the index injury were known, our data do not include sports played at the time of the second ACL injury or what level of sports participation the patient engaged in at that time. In addition, the current model does not distinguish the risk based on ACL graft tears in the surgical limb or ACL injuries in the contralateral limb. It is possible that specific classification methods, predictive factors, or the relative weight of these predictors may differ based on the injury type. While limitations in dataset size prohibit the exploration of this within the current study, this represents an area of future opportunity as this work progresses. While the breadth of variables utilized within model development was a strength of this project, the predictor variables currently cannot account for other anatomic (eg, tibial slope, femoral notch width), psychological (eg, ACL–Return to Sport after Injury scale score), neuromuscular (eg, limb biomechanics), or surgical (eg, with or without lateral extra-articular tenodesis) factors that were not available within our current dataset. The inclusion of these measures may improve the accuracy of classification and the identification of modifiable risk factors in our system. Efforts to expand the data inputs for our model are currently underway.

Missing data is a common problem encountered in machine learning applications utilizing real-world datasets. The mean imputation methodology employed within the current study has been shown to be highly accurate for handling missing data within medical datasets. However, data imputation does create the risk that the model will be overfit to the original dataset and limit its generalizability to new data. Further validation using an external dataset is needed to verify generalizability. It should also be noted that the timing of functional performance testing (strength and hop tests) was not controlled, and variability in the timing of data collection may have influenced the model.

Finally, continued work is needed to refine the recommendations of the ACL-RRP model within the output for suggested corrections to address the specified modifiable risk factors. To this point, machine learning models continually evolve as more training data allow for the identification of new patterns in data. As work on the ACL-RRP model continues to evolve, we believe that this limitation will be resolved with the acquisition of more training data.

## Conclusion

The ACL-RRP model utilized a unique interpretable machine learning approach to (1) determine a patient’s overall risk of sustaining a repeat ACL injury, (2) differentiate between modifiable and nonmodifiable risk factors, and (3) generate a report to clinicians ranking the modifiable risk factors by ease of modification and influence on the risk. The inclusion of rehabilitation and physical performance data in prediction models may enhance the accuracy and clinical utility of the ACL-RRP model. While this work should be considered preliminary, the high level of accuracy seen within our results supports continued research on expanding predictor variables, external validation, and developing a user interface for widespread adoption and prospective investigation.

## Supplemental Material

sj-docx-1-ojs-10.1177_23259671251329355 – Supplemental material for Precision Rehabilitation After Youth Anterior Cruciate Ligament Reconstruction: Individualized Reinjury Risk Stratification and Modifiable Risk Factor Identification to Guide Late-Phase Rehabilitation Supplemental material, sj-docx-1-ojs-10.1177_23259671251329355 for Precision Rehabilitation After Youth Anterior Cruciate Ligament Reconstruction: Individualized Reinjury Risk Stratification and Modifiable Risk Factor Identification to Guide Late-Phase Rehabilitation by Elliot M. Greenberg, Amanda Watson, Kimberly Helm, Kevin Landrum, J. Todd R. Lawrence and Theodore J. Ganley in Orthopaedic Journal of Sports Medicine
