# Supplementary material for: Precision Rehabilitation After Youth Anterior Cruciate Ligament Reconstruction: Individualized Reinjury Risk Stratification and Modifiable Risk Factor Identification to Guide Late-Phase Rehabilitation
Source: Orthop J Sports Med. 2025 Apr 11;13(4):23259671251329355. doi: 10.1177/23259671251329355 (PMC12032490; doi:10.1177/23259671251329355)
Supplement: sj-docx-1-ojs-10.1177_23259671251329355 – Supplemental material for Precision Rehabilitation After Youth Anterior Cruciate Ligament Reconstruction: Individualized Reinjury Risk Stratification and Modifiable Risk Factor Identification to Guide Late-Phase Rehabilitation [file sj-docx-1-ojs-10.1177_23259671251329355.docx]

APPENDIX

Labeling Functions for Each Risk Factor Stratifying Between High, Low, and Unknown Risk

| **Feature** | **High** | **Low** | **Unknown** |
| --- | --- | --- | --- |
| Age at Surgery(months) | (x >=144) & (x <=204) | (x < 144) \| (x > 204) |  |
| Age at Return to Sport | (x >= 13) & (x < 18) | (x >= 18) | (x < 13) |
| Delay to Surgery | (x <= 21) |  | (x > 21) |
| Time to Release for Activity | (x <= 319) | (x > 319) |  |
| Time to repeat ACL tear (days) | (x <= 730) & (x >= 365) | (x >= 1095) | (x < 365) \| ((x > 730) & (x <= 1095)) |
| Sex1=male2=female | (x == 2) | (x == 1) |  |
| Age & Gender | (x == 1)If age >12 female higher risk If age <12 male | (x == 0)If >12 years-old Males  If <12 years-old females |  |
| BMI | (x >= 35) | (x <= 20) | (x > 25) & (x < 35) |
| Meniscus resection (old)0=no1=yes | (x == 1) | (x == 0) |  |
| Meniscus tear (old) | (x == 1) | (x == 0) |  |
| 180 deg/s LSI QUADS (calc)_final | (x <= 90) | x > 90 |  |
| Involved Limb Hams/Quad ratio_final | (x < 60) \| (x > 65) | (x >= 60) & (x <= 65) |  |
| Uninvolved Limb Hams/Quads Ratio_final | (x < 60) \| (x > 65) | (x >= 60) & (x <= 65) |  |
| Involved limb peak torque normalized to body weight QUADS_final | (x <2.2) \| (x>2.8) | (x>=2.3) & (x <= 2.7) |  |
| Involved limb peak torque normalized to body weight HAMS_final | Value greater than 1 standard deviation below the mean of the entire cohort  Value greater than 1 standard deviation above the mean of the cohort | Values within 1 standard deviation of the mean |  |
| Total Work LSI QUADS_final | (x < 90) | (x >= 90) & (x <= 100) | (x > 100) |
| Total Work LSI HAMS_final | (x < 90) | (x >= 90) & (x <= 100) | (x > 100) |
| Involved limb total work QUADS_final | Value greater than 1 standard deviation below the mean of the entire cohort  Value greater than 1 standard deviation above the mean of the cohort | Values within 1 standard deviation of the mean |  |
| uninvolved limb peak torque normalized to body weight QUADS_final | (x <2.2) \| (x>2.8) | (x>=2.3) & (x <= 2.7) |  |
| uninvolved limb peak torque normalized to body weight HAMS_final | Value greater than 1 standard deviation below the mean of the entire cohort  Value greater than 1 standard deviation above the mean of the cohort | Values within 1 standard deviation of the mean |  |
| Single Leg Hop LSI_final | (x < 90) | (x >= 90) & (x <= 100) | (x > 100) |
| Triple Hop LSI_final | (x < 90) | (x>=90) & (x <=100) | (x > 100) |
| Vertical Hop LSI_final | (x < 90) | (x>=90) & (x <=100) | (x > 100) |
| Involved Limb Triple Hop Distance (cm) Normalized to Body Height (cm)_final | ((x > 2.5) & (x <= 3)) \| (x < 1.9) | (x >= 1.9) & (x <= 2.5) | (x > 3) |
| Involved Limb Vertical Jump Normalized to body height_final | (x < .1) | (x > .15) | (x >= .1) & (x <= .15) |
| Sport Played at Injury Codes | (x == 1)Basketball Field Hockey Football Lacrosse Rugby Skiing Soccer Volleyball | (x == 3)Baseball Softball Running Swimming Injuries unrelated to sports | (x == 2)Cheerleading Gymnastics Ice Hockey Racquet sports (tennis, squash) Snowboard Wrestling |
